# A novel guiding device for puncture localization in percutaneous transforaminal endoscopic discectomy

**DOI:** 10.1186/s13018-025-06022-5

**Published:** 2025-06-24

**Authors:** Huang Yong, Hang Shi, Rong Xue, Guoping Zhu, Yuqing Chen

**Affiliations:** 1 The Department of Orthopedics, The Affiliated Xinghua People’s Hospital, Medical School of Yangzhou University, Xinghua, Jiangsu China; 2 Department of Spine Surgery, Zhongda Hospital, Nanjing, China; 3 School of Medicine, Southeast University, Nanjing, China

**Keywords:** Transforaminal, Discectomy, Guiding device, Puncture, Fluoroscopy

## Abstract

**Background:**

Conventional fluoroscopy-based positioning techniques for percutaneous transforaminal endoscopic discectomy (PTED) depend heavily on the operator’s spatial cognition and surgical expertise, typically necessitating multiple puncture attempts under iterative radiographic monitoring, which inherently involves substantial risks of iatrogenic injury and cumulative radiation exposure. This study aims to develop a simple and practical novel puncture guiding device for PTED and apply it in PTED surgery.

**Methods:**

A novel guiding device for assisting puncture localization in PTED was developed. Patients scheduled for PTED were randomly divided into two groups: Group A and Group B, with 30 cases in each group. Group A used the novel guiding device for puncture localization in PTED, while Group B used the traditional fluoroscopic puncture method. The subsequent endoscopic surgical procedures were the same for both groups. The basic data of the patients, intraoperative puncture localization evaluation indicators (number of punctures, number of fluoroscopies, puncture localization time, surgical time), and surgical efficacy evaluation indicators were recorded and compared.

**Results:**

The number of punctures in Group A and Group B were 4.40 ± 0.89 and 7.93 ± 2.27, with Group A having significantly fewer punctures than Group B (*P* < 0.05). The number of fluoroscopies in the two groups were 13.03 ± 1.13 and 20.70 ± 3.34, respectively, with Group A having significantly fewer fluoroscopies than Group B (*P* < 0.05). The puncture localization time in the two groups were 34.57 ± 5.52 min and 43.00 ± 6.38 min, respectively, with Group A having a shorter puncture localization time than Group B (*P* < 0.05). The total surgical time in the two groups were 91.00 ± 10.70 min and 99.67 ± 10.08 min, respectively, with Group A having a shorter total surgical time than Group B (*P* < 0.05). There were no significant statistical differences in surgical efficacy evaluation indicators between the two groups (*P* > 0.05).

**Conclusion:**

The novel guiding device has the advantages of being easy to use, fast and accurate in puncture, and having a high success rate. Compared with the traditional fluoroscopic puncture method, the novel guiding device can reduce the difficulty of puncture localization in PTED, improve puncture accuracy, and reduce the number of punctures, fluoroscopies, and puncture localization time.

## Introduction

Percutaneous transforaminal endoscopic discectomy (PTED) offers several advantages, including the ability to be performed under local anesthesia, minimal tissue damage, reduced bleeding, rapid postoperative recovery, and excellent surgical outcomes [1–4]. Safe and accurate puncture localization is a critical step in this procedure, requiring surgeons to possess strong spatial imagination and extensive surgical experience. The learning curve for beginners is steep [5, 6]. Traditional fluoroscopy-guided puncture localization often involves repeated punctures under continuous fluoroscopic guidance, which increases the risk of vascular, neural, and visceral injuries [7]. Additionally, repeated fluoroscopic exposure raises the likelihood of radiation-related diseases, with excessive radiation exposure potentially leading to severe conditions such as cataracts, leukemia, skin erythema, and thyroid cancer [8–10]. Even with the current maturity of percutaneous transforaminal endoscopic discectomy techniques, reducing the difficulty of puncture localization, minimizing puncture-related injuries, and lowering radiation exposure remain clinically significant. To address these challenges, we previously developed a novel guiding device with a simple structure and user-friendly design. Initial animal model experiments demonstrated promising results in assisting transforaminal puncture localization [11]. However, animal model results cannot fully represent clinical outcomes, as the surgical environment is more complex. Beyond puncture localization metrics, surgical safety and efficacy must also be considered. This study applies the novel guiding device to the puncture localization during PTED and compares it with the traditional fluoroscopic puncture method to evaluate the actual clinical efficacy of the novel guiding device.

## Methods

### Design and fabrication of the novel guiding device

**Fig. 1:**
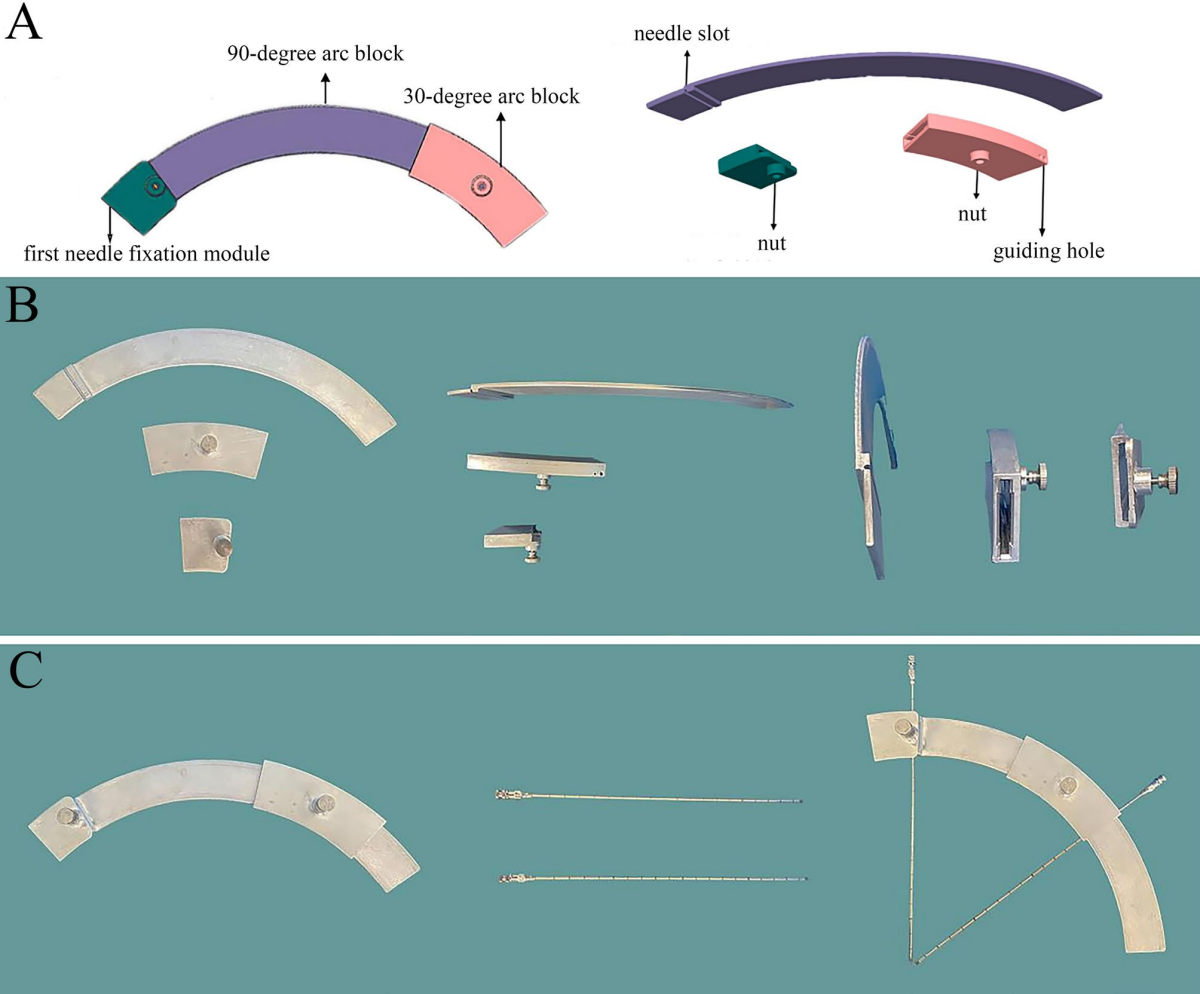
Design and physical images of the novel guiding device. **A**: Three-dimensional structural design diagram of the guiding device. **B**: Physical image of the guiding device made of aluminum alloy. **C**: Assembly of the guiding device with the puncture needles

The three-dimensional structure of the novel guiding device was designed using “UG” (Unigraphics NX) software. The device is based on the principle of concentric spherical positioning and consists of three main components: a 90-degree arc block, a 30-degree arc block, and a first needle fixation module. The 30-degree arc block is hollow and can slide over the 90-degree arc block (Fig. 1A). In collaboration with a machining company, the device was fabricated from lightweight aluminum alloy using Beijing Jingdiao CNC machines (Fig. 1B). The outer radius of the 90-degree arc block is 180 mm, and the inner radius is 150 mm. One end of the 90-degree arc block features a needle slot (2.12 mm in diameter) for the first puncture needle. The first needle fixation module can be inserted into the needle slot end of the 90-degree arc block, and the first puncture needle is secured in place using a nut. The 30-degree arc block can be slid over the other end of the 90-degree arc block, allowing for concentric movement. It includes a guiding hole (2.12 mm in diameter) for the second puncture needle and a nut to fix the relative positions of the two arc blocks. The device requires two puncture needles for operation. To prevent needle deformation, 14G (2.1 mm in diameter) puncture needles with a length of 200 mm were selected (Fig. 1C).

### Principle of using the novel guiding device

**Fig. 2:**
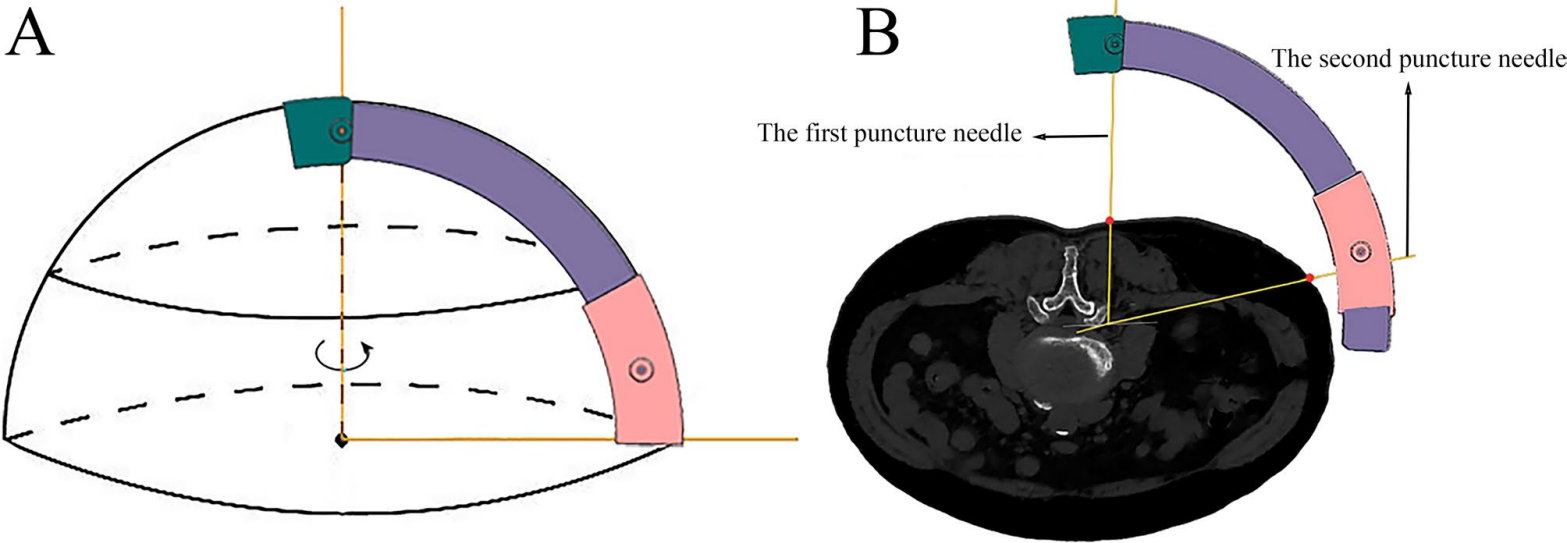
Schematic diagram of the working principle of the novel guiding device. **A**: Concentric sphere positioning principle: If the arc guide is rotated with the first puncture needle as the central axis, the outer arc of the 90-degree arc block will form a virtual hemisphere. By rotating the 90-degree arc block and simultaneously sliding the 30-degree arc block, the second puncture needle can reach the center of the sphere through the guiding hole at any radius. **B**: Indirect puncture principle for lumbar transforaminal approach: The first puncture needle is inserted from the posterior direction along the lateral side of the superior articular process until it reaches the ventral edge. The first puncture needle is then connected to the guiding device, with its tip positioned at the center of the sphere. The second puncture needle is aligned with the posterolateral puncture point and punctured through the guiding hole. According to the concentric sphere positioning principle, the second needle will converge with the first needle at the lateral and ventral edges of the superior articular process. Further advancement of the needle will enter the intervertebral foramen and ultimately reach the posterior region of the herniated disc

Concentric sphere positioning principle: The first puncture needle is fixed in the needle slot of the 90-degree arc block. When the puncture needle intersects the outer arc at a scale of 180 mm (the radius length), the needle tip is positioned at the center of the sphere. By rotating the 90-degree arc block with the first puncture needle as the central axis, the outer arc forms a virtual hemisphere. The second puncture needle is inserted into the guiding hole of the 30-degree arc block. By rotating the 90-degree arc block and simultaneously sliding the 30-degree arc block, the second puncture needle can reach the center of the sphere through the guiding hole at any radius and converge with the first puncture needle at the center (Fig. 2A).

Indirect puncture principle for lumbar transforaminal approach: First, the first puncture needle is inserted from the posterior direction along the lateral side of the lumbar superior articular process until it reaches the ventral edge of the superior articular process (SAP). The first puncture needle was abducted to match the SAP angle rather than maintaining perpendicularity, ensuring lateral SAP surface proximity. The first puncture needle is then connected to the needle slot of the novel guiding device, ensuring the needle tip is positioned at the center of the sphere. The second puncture needle is inserted into the guiding hole of the 30-degree arc block. By rotating the 90-degree arc block and sliding the 30-degree arc block simultaneously, the second puncture needle is aligned with the posterolateral puncture point and punctured through the guiding hole. According to the concentric sphere positioning principle, the second puncture needle will converge with the first puncture needle at the lateral and ventral edge of the superior articular process (i.e., the posterior border of the lower half of the intervertebral foramen’s outer opening). Further advancement of the needle will enter the intervertebral foramen and ultimately reach the posterior region of the herniated disc (Fig. 2B). This indirect puncture method transforms a technically challenging lumbar transforaminal puncture into two simpler punctures.

### Clinical study design

A prospective randomized controlled clinical trial was conducted, enrolling patients who underwent percutaneous transforaminal endoscopic discectomy at our hospital from June 2021 to December 2023. Patients scheduled for percutaneous transforaminal endoscopic discectomy were randomly divided into two groups: Group A and Group B, with 30 cases in each group. Group A used the novel guiding device for puncture localization in PTED, while Group B used the traditional fluoroscopic puncture method. The subsequent endoscopic surgical procedures were the same for both groups. All surgeries were performed by the same experienced surgeon. The basic data, intraoperative puncture localization evaluation indicators, and surgical efficacy evaluation indicators were compared between the two groups. This study was approved by the Medical Ethics Committee of Xinghua People’s Hospital, and all patients signed informed consent forms to participate in the study.

Inclusion criteria: (1) Age between 18 and 70 years; (2) Single-level lumbar disc herniation (central, paracentral, or foraminal type) as the responsible segment; (3) Unilateral lower limb radicular pain, with or without low back pain or buttock pain; (4) Failure or worsening of symptoms after 6 weeks of conservative treatment; (5) Agreement to participate in the study and signing of the informed consent form; (6) Follow-up period of 6 months.

Exclusion criteria: (1) Extreme lateral disc herniation; (2) Two or more responsible segments; (3) Significant lumbar spinal stenosis, lumbar instability, or spondylolisthesis; (4) Isolated low back pain or buttock pain; (5) Cauda equina syndrome; (6) Previous spinal surgery; (7) Spinal infection, metastatic tumors, etc.

Withdrawal criteria: Patients who signed the informed consent form and entered the randomized trial but withdrew from the trial for any reason at any time, without completing the protocol and the required observation period tasks, were considered withdrawal cases.

### Evaluation indicators

(1) Basic data: age, gender, body mass index (BMI), level of disc herniation, and type of disc herniation.

(2) Intraoperative puncture localization evaluation indicators: number of punctures, number of fluoroscopies, puncture localization time, and surgical time. The number of punctures is defined as the total number of times the puncture direction needs to be changed and re-punctured based on fluoroscopy results. For the guiding device group, the number of punctures is the sum of punctures at two sites. The number of fluoroscopies refers to the total number of fluoroscopies from the initial marking of the puncture point under fluoroscopy to the completion of working cannula placement. Puncture localization time refers to the total time from marking the puncture point to completing the placement of the working cannula. Surgical time refers to the total time from marking the puncture point to the end of the surgery.

(3) Surgical efficacy evaluation indicators: Preoperative low back pain visual analog scale (VAS) score, leg pain VAS score, and oswestry disability index (ODI) were recorded for both groups. Follow-ups were conducted at 1 month, 3 months, and 6 months postoperatively. At the final 6-month follow-up, the low back pain VAS score, leg pain VAS score, ODI, and modified Macnab grading were recorded for both groups. Surgical complications were recorded. Intraoperative complications mainly included visceral injury, nerve root injury, dural tear, cerebrospinal fluid leakage, severe bleeding, and intraoperative head and neck pain. Postoperative complications mainly included infection, cerebrospinal fluid leakage, deep hematoma, postoperative recurrence, and reoperation. Reoperation was defined as a second surgery at the same level and side as the initial surgery. The reason for reoperation was determined based on MRI findings.

### Surgical procedure

#### Surgical procedure in Group A (using a case of L4/5 left paracentral disc herniation as an example)

**Fig. 3:**
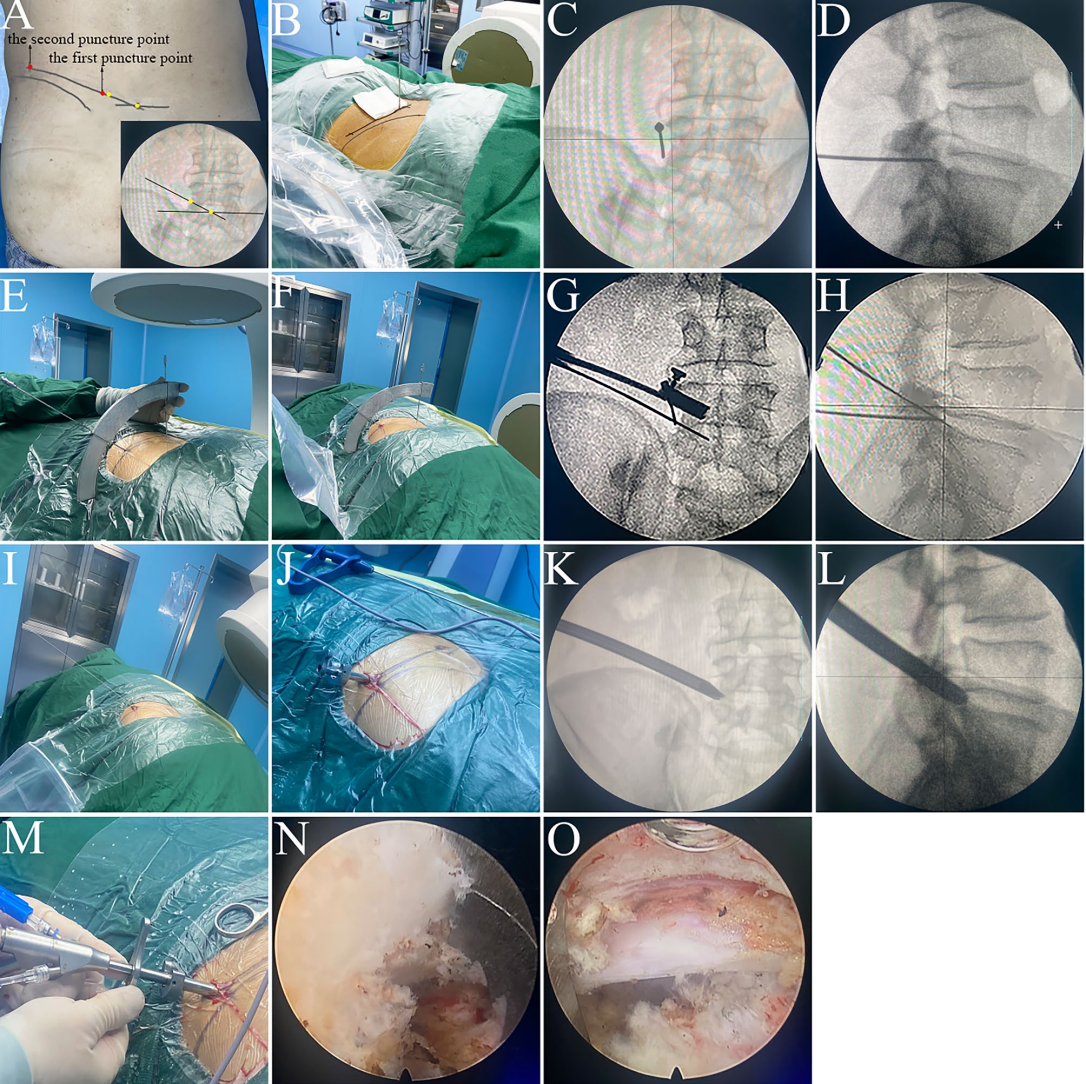
Surgical procedure in Group A. **A**: Marking two puncture points under fluoroscopy. **B**: The puncture of the first puncture needle. **C**: AP fluoroscopy: the first puncture needle tip positioned lateral to the superior articular process. **D**: Lateral fluoroscopy: the first puncture needle aligned with the ventral border of the superior articular process. **E**: Align the second puncture needle with the second puncture point. **F**: The second puncture needle was gradually advanced along the guiding hole. **G**: AP fluoroscopy showed the second puncture needle located between the medial edge of the pedicle and the spinous process. **H**: Lateral fluoroscopy showed the second puncture needle located at the posterior edge of the intervertebral disc. **I**: A guidewire was inserted through the inner core hole of the second puncture needle. **J**: Placement of the working cannula. **K, L**: AP and lateral fluoroscopy confirmed the position of the working cannula. **M**: Connect endoscope. **N**: Foraminal enlargement. **O**: Endoscopic surgical procedures

(1) Marking two puncture points under anteroposterior (AP) fluoroscopy: The patient was placed in a prone position. Under AP fluoroscopy, two puncture points were marked. The first puncture point was located 0.5 cm lateral to the dorsal projection of the tip of the superior articular process of the lower vertebral body, the second puncture point was located on the line connecting the midpoint of the superior endplate of the lower vertebral body and the tip of the superior articular process, 8 ~ 14 cm lateral to the midline (generally 8 ~ 10 cm for L3/4, 10 ~ 12 cm for L4/5, and 12 ~ 14 cm for L5/S1) (Fig. 3A).

(2) The puncture of the first puncture needle: The first puncture needle was inserted from posterior to anterior at the initial puncture point under local anesthesia, first contacting the lateral edge of the superior articular process, then slightly sliding downward along the lateral edge. (Fig. 3B). The position of the needle was adjusted under lateral fluoroscopy to ensure proper depth and alignment. The final position of the needle should be confirmed under AP fluoroscopy as lateral to the superior articular process and under lateral fluoroscopy as ventral to the superior articular process (Fig. 3C and D).

(3) The puncture of the second puncture needle: The first puncture needle was connected to the needle slot of the guiding device and fixed with a nut at the 180 mm mark, ensuring the needle tip is at the center of the virtual sphere. The second puncture needle was inserted into the guide hole of the 30-degree arc block. While holding the first puncture needle and guiding device steady, the arc block was rotated and slid along the axis of the first puncture needle to align the tip of the second puncture needle with the second puncture point (Fig. 3E). The relative positions of the two arc blocks were then fixed with nuts. The second puncture needle was gradually inserted along the guiding hole under the local anesthetic. Based on the concentric sphere positioning principle, the second puncture needle would converge with the first puncture needle at the virtual sphere center, which was located lateral and anterior to the superior articular process. The second puncture needle converged with the first needle at the virtual sphere center (the lateral and anterior aspect of the superior articular process). The second needle was then slowly advanced approximately 15 mm further, entering the intervertebral foramen and reaching the posterior margin of the intervertebral disc. (Fig. 3F). Fluoroscopy confirmed the position of the second puncture needle: under AP fluoroscopy, it should be located between the medial edge of the pedicle and the spinous process (Fig. 3G), and under lateral fluoroscopy, it should be located at the posterior edge of the intervertebral disc (Fig. 3H).

(4) Guidewire insertion and working cannula placement: A guidewire was inserted through the inner core hole of the second puncture needle. The guiding device and puncture needles were then removed, leaving only the guidewire in place (Fig. 1I). After confirming the correct position of the guidewire under fluoroscopy, the tract was dilated stepwise, and the working cannula was placed (Fig. 3J). The position of the working cannula was confirmed under AP and lateral fluoroscopy (Fig. 3K and L).

(5) Endoscopic surgical procedures: The endoscopic system was connected (Fig. 3M). Under endoscopic visualization, a trephine was used to remove the ventral portion of the superior articular process to enlarge the intervertebral foramen (Fig. 3N). The endoscope was advanced into the spinal canal, and the following procedures were performed: removal of the herniated nucleus pulposus, decompression of the nerve root, hemostasis using a radiofrequency probe, and shaping of the posterior longitudinal ligament and annulus fibrosus (Fig. 3O).

#### Surgical procedure in Group B

The patient was placed in a prone position with hips flexed, and a pad was used to elevate the abdomen. The puncture point was first marked under anteroposterior (AP) fluoroscopy (consistent with the localization method used in the previous two groups): on the line connecting the midpoint of the superior endplate of the lower lumbar vertebra and the tip of the superior articular process, 8 ~ 14 cm lateral to the midline (generally 8 ~ 10 cm for L3/4, 10 ~ 12 cm for L4/5, and 12 ~ 14 cm for L5/S1). After routine disinfection and draping, the skin and subcutaneous tissue at the puncture site were anesthetized with 1% lidocaine. Under fluoroscopic guidance, a 16G puncture needle was used for the transforaminal puncture, with deep anesthesia achieved using 0.5% lidocaine. The target for the puncture was as follows: under AP fluoroscopy, the needle tip should be located at the tip of the superior articular process, under lateral fluoroscopy, the needle tip should be located at the ventral edge of the tip of the superior articular process. Once the needle reached the target, a guidewire was inserted. The tract was then dilated stepwise using dilators along the guidewire, followed by the placement of the working cannula and endoscope. The subsequent endoscopic surgical procedures were consistent with those performed in Group A.

### Postoperative management

Postoperative patients were required to rest in bed for more than 6 h, after which they could gradually begin ambulation with lumbar support. After suture removal, patients were encouraged to perform lumbar muscle exercises to promote functional recovery. Bending and weight-bearing were prohibited for 6 weeks following the surgery. The lumbar support was typically removed after 6 weeks.

### Statistical methods

Statistical analysis was performed using SPSS 25.0 software. Categorical data were expressed as counts and compared using Pearson’s chi-square test or Fisher’s exact test. Continuous data were expressed as mean ± standard deviation. Normality tests were conducted for all continuous data. For data conforming to a normal distribution, the independent samples t-test was used for comparison between groups. For data not conforming to a normal distribution, the Mann-Whitney U test was applied. A *p*-value of < 0.05 was considered statistically significant.

## Results

### Baseline characteristics

There were no statistically significant differences in baseline characteristics such as age, gender, body mass index (BMI), and surgical level between the two groups (*P* > 0.05) (Table 1).

**Table 1:** Comparison of baseline characteristics between the two groups

Characteristics	Group A(*n* = 30)	Group B(*n* = 30)	*P*-value
Age (years)	43.90 ± 8.49	45.03 ± 8.04	0.598^#^
Gender (male/female)	16/14	18/12	0.411^+^
BMI(kg/m^2^)	23.67 ± 1.95	24.09 ± 1.08	0.387^*^
Surgical level	-	-	0.846^+^
L3/4	1	2	
L4/5	24	20	
L5/S1	5	8	
Herniation location	-	-	0.832^+^
Paracentral	19	21	
Central	8	7	
Foraminal	3	2	

Values are presented as mean ± SD or number. # t-test, ∗Mann–Whitney U test, + Pearson’s chi-square test or Fisher’s exact test

### Intraoperative puncture localization metrics

All cases successfully underwent percutaneous endoscopic lumbar discectomy. The comparison of intraoperative puncture localization evaluation indicators between the two groups is shown in Table 2. The number of punctures in the Group A and Group B were 4.40 ± 0.89 and 7.93 ± 2.27, respectively. The Group A had fewer punctures than Group B, and the difference was statistically significant (*P* < 0.05). The number of fluoroscopies in the two groups were 13.03 ± 1.13 and 20.70 ± 3.34, respectively. The Group A had fewer fluoroscopies than the Group B, and the difference was statistically significant (*P* < 0.05). The puncture localization times in the two groups were 34.57 ± 5.52 min and 43.00 ± 6.38 min, respectively. The Group A had a shorter puncture localization time than the Group B, and the difference was statistically significant (*P* < 0.05). The surgical times in the two groups were 91.00 ± 10.70 min and 99.67 ± 10.08 min, respectively. The Group A had a shorter surgical time than the Group B, and the difference was statistically significant (*P* < 0.05).

**Table 2:** Comparison of intraoperative puncture positioning metrics between the two groups

Metrics	Group A(*n* = 30)	Group B(*n* = 30)	*P*-value
Number of punctures	4.40 ± 0.89	7.93 ± 2.27	< 0.001^*^
Number of fluoroscopies	13.03 ± 1.13	20.70 ± 3.34	< 0.001^*^
Puncture positioning time(min)	34.57 ± 5.52	43.00 ± 6.38	< 0.001^*^
Total surgical time (min)	91.00 ± 10.70^*#^	99.67 ± 10.08	< 0.001^*^

Values are presented as mean ± SD. ∗Mann–Whitney U test

### Surgical efficacy evaluation metrics

The preoperative VAS scores for back pain and leg pain, as well as the ODI index in the Group A, were 5.80 ± 0.66, 6.17 ± 0.65, and 57.13 ± 6.72%, respectively. At the 6-month postoperative follow-up, the VAS scores for back pain and leg pain, ODI index, and the excellent/good rate of the modified MacNab criteria were 1.23 ± 0.86, 1.33 ± 0.96, 12.63 ± 8.37%, and 90.00%, respectively. In the Group B, the preoperative VAS scores for back pain and leg pain, as well as the ODI index, were 6.10 ± 0.80, 6.13 ± 0.73, and 60.13 ± 7.33%, respectively. At the 6-month postoperative follow-up, the VAS scores for back pain and leg pain, ODI index, and the excellent/good rate of the modified MacNab criteria were 1.33 ± 0.61, 1.33 ± 0.92, 13.93 ± 7.46%, and 93.33%, respectively. There were no statistically significant differences in the preoperative VAS scores for back pain and leg pain, as well as the ODI index, between the two groups (*P* > 0.05). Similarly, there were no statistically significant differences in the VAS scores for back pain and leg pain, ODI index, and the excellent/good rate of the modified MacNab criteria at the 6-month postoperative follow-up between the two groups (*P* > 0.05). The improvement values in the VAS scores for back pain and leg pain, as well as the ODI index, from preoperative to 6 months postoperative were not significantly different between the two groups (*P* > 0.05). None of the cases in either group experienced severe complications such as visceral injury, permanent nerve root injury, cerebrospinal fluid leakage, significant bleeding, or postoperative infection. In both groups, one case experienced intraoperative head and neck pain, which resolved by the next day. In the Group A, there were 3 cases of postoperative recurrence, 2 of which underwent open surgery after failed conservative treatment. In the Group B, there were 2 cases of postoperative recurrence, 1 of which improved with conservative treatment, and 1 required open surgery after failed conservative treatment. There were no statistically significant differences in surgical complications between the two groups (*P* > 0.05). The comparison of surgical efficacy evaluation metrics between the two groups is detailed in Table 3.

**Table 3:** Comparison of efficacy evaluation metrics between the two groups

Metrics	Group A(*n* = 30)	Group B(*n* = 30)	*P*-value
VAS score for back pain			
Preoperative	5.80 ± 0.66	6.10 ± 0.80	0.127^*^
6 months postop	1.23 ± 0.86	1.33 ± 0.61	0.689^*^
Improvement score	4.57 ± 1.14	4.77 ± 1.04	0.535^*^
VAS score for leg pain			
Preoperative	6.17 ± 0.65	6.13 ± 0.73	0.865^*^
6 months postop	1.33 ± 0.96	1.33 ± 0.92	0.878^*^
Improvement score	4.83 ± 1.15	4.80 ± 1.16	0.810^*^
ODI Index (%)			
Preoperative	57.13 ± 6.72	60.13 ± 7.33	0.139^*^
6 months postop	12.63 ± 8.37	13.93 ± 7.46	0.095^*^
Improvement	44.50 ± 11.49	46.20 ± 10.68	0.705^*^
MacNab grading system			0.921^+^
Excellent/good	15/11(90.00%)	15/13(93.33%)	
Fair/poor	2/1	1/1	
Complications			0.892^+^
Head and neck pain	1	1	
Recurrence	3	2	
Reoperation	2	1	

Values are presented as mean ± SD or number. ∗Mann–Whitney U test, + Pearson’s chi-square test or Fisher’s exact test

## Discussion

Currently, researchers have developed various auxiliary puncture technologies, primarily including imaging systems [12], ultrasound volume navigation [13, 14], electromagnetic navigation [15, 16], optical navigation [17–19], surgical robots [20], 3D-printed guides [21], and guide devices [22]. Among these, the guiding device is a low-cost, reusable, simple, and practical auxiliary puncture technology. This study developed a novel guiding device and applied it to puncture localization in PTED. The results showed that, compared to the traditional fluoroscopy-guided puncture method, the novel guiding device reduced the difficulty of puncture localization in PTED, improved puncture accuracy, and simultaneously decreased the number of punctures and fluoroscopies, as well as shortened puncture localization time and total surgical time. There were no statistically significant differences in the evaluation indicators of surgical efficacy, such as VAS scores for low back and leg pain, ODI index, modified Macnab grading, and surgical complications, between the two groups. This study provides a fast and accurate new method for puncture localization in PTED.

The novel guiding device is an arc-shaped device based on the concentric sphere positioning principle. It has a simple structure, compact size, and is easy to carry and use. The working principle of the novel guiding device is clear, employing an indirect puncture and positioning method, which is similar to the use of a locator in anterior cruciate ligament (ACL) reconstruction. In ACL reconstruction, the hook end of the locator is first used to determine the insertion point of the ACL, and then a Kirschner wire is percutaneously inserted from the proximal tibia using the locator, directly reaching the hook end position. Similarly, in the novel guiding device-assisted puncture, the first puncture needle is inserted posteriorly along the lateral edge of the superior articular process, easily reaching the lateral and ventral edge of the superior articular process. Then, the second puncture needle is inserted from the posterolateral marked puncture point with the assistance of the guiding device. According to the concentric sphere positioning principle, the second puncture needle will converge with the first needle at the lateral and ventral edge of the superior articular process, and further insertion will reach the target position behind the intervertebral disc. The puncture of the first needle is critical. If necessary, the position of the first puncture needle can be adjusted under lateral fluoroscopy to a precise location on the lateral side of the superior articular process (e.g., the tip of the superior articular process). In this study, no cases of exiting nerve root injury or significant bleeding occurred. Compared to the traditional fluoroscopic puncture method, although the novel guiding device-assisted puncture involves an additional posterior puncture, the second puncture needle can achieve successful puncture in one attempt via the transforaminal approach, thereby improving the safety of puncture localization in PTED while significantly increasing the puncture speed. The two needle punctures required by the novel guiding device might theoretically increase intraoperative pain scores. During the procedure, we routinely asked patients about their pain perception, and most reported that the posterior puncture was tolerable. However, specific pain scores were not systematically recorded. We believe that the pain associated with the posterior puncture can be compared to that of percutaneous vertebroplasty (PVP) punctures. Under local anesthesia, when the puncture does not involve bone penetration, the perceived pain is generally mild. As long as sufficient local anesthesia is administered, the additional puncture is unlikely to cause a clinically meaningful increase in intraoperative pain.

The straightness of the puncture needle is closely related to the accuracy of the novel guiding device-assisted puncture. Although 14G puncture needles are less prone to bending, care must still be taken to avoid bending during application. The following points should be noted: Before puncture, assemble the guiding device with the two puncture needles and routinely check the needle shape, replacing any bent needles promptly. After connecting the puncture needle to the guiding device, stabilize the guiding device with one hand to avoid bending or displacement of the puncture needle. Avoid forceful insertion to prevent needle bending. For the L5/S1 segment, ensure the puncture needle is not too close to the iliac crest to avoid excessive resistance that could cause needle bending. Similar to traditional fluoroscopy-guided puncture, using the novel guiding device-assisted puncture also requires a spatial imagination of lumbar anatomy.

Fan Guoxin et al. reported an arc-shaped locator independently designed based on the concentric sphere positioning principle. This device consists of a movable support part, a three-dimensional joystick adjustment part, and an arc angle-guided puncture part. It requires marking the projection points of the puncture target on the dorsal and lateral skin under standard AP and lateral fluoroscopy. If the C-arm is not positioned vertically, errors may occur during surface marking, sometimes necessitating re-marking of the projection points [22]. Compared to the aforementioned guiding device, the novel guiding device combines the concentric sphere positioning principle with the indirect puncture principle, eliminating the need for accurate marking of the herniated nucleus pulposus projection under fluoroscopy. In practice, the puncture of the first needle replaces the step of marking the nucleus projection under fluoroscopy, reducing reliance on fluoroscopy and theoretically increasing the success rate of puncture.

In this study, the novel guiding device was applied to assist puncture localization in PTED. Compared to the traditional fluoroscopy-guided puncture method, the novel guiding device reduced the difficulty of puncture and positioning, improved puncture accuracy, decreased the number of punctures and fluoroscopies, and shortened puncture localization time, thereby reducing patient puncture injury and radiation exposure risks. This study has the following limitations: The lack of radiation dosimetry data (despite recording fluoroscopy counts) represents a methodological limitation. The number of cases in the clinical trial is small, which may affect the generalizability of our results. Future multicenter trials with larger sample sizes are needed to validate the generalizability of the results.

## Conclusion

The novel guiding device has the advantages of being easy to use, fast and accurate in puncture, and having a high success rate. Compared with the traditional fluoroscopic puncture method, the novel guiding device can reduce the difficulty of puncture localization in PTED, improve puncture accuracy, and reduce the number of punctures, fluoroscopies, and puncture localization time. The novel guiding device is expected to reduce the risk of puncture injury and radiation exposure for patients, as well as improve the learning curve for beginners, making it a simple and practical new technology for assisting puncture localization in PTED.

## Data Availability

No datasets were generated or analysed during the current study.
